# Digital Eye Strain Monitoring for One-Hour Smartphone Engagement Through Eye Activity Measurement System

**DOI:** 10.3390/jemr18040034

**Published:** 2025-08-05

**Authors:** Bhanu Priya Dandumahanti, Prithvi Krishna Chittoor, Murali Subramaniyam

**Affiliations:** 1 Department of Computing Technologies, SRM Institute of Science and Technology, Chennai 603203, India; dandumap1@srmist.edu.in; 2 Engineering Product Development Pillar, Singapore University of Technology and Design, Singapore 487372, Singapore; 3 Department of Mechanical Engineering, SRM Institute of Science and Technology, Chennai 603203, India

**Keywords:** blink rate, digital eyestrain, pupil dilation, smartphone usage, visual fatigue

## Abstract

Smartphones have revolutionized our daily lives, becoming portable pocket computers with easy internet access. India, the second-highest smartphone and internet user, experienced a significant rise in smartphone usage between 2013 and 2024. Prolonged smartphone use, exceeding 20 min at a time, can lead to physical and mental health issues, including psychophysiological disorders. Digital devices and their extended exposure to blue light cause digital eyestrain, sleep disorders and visual-related problems. This research examines the impact of 1 h smartphone usage on visual fatigue among young Indian adults. A portable, low-cost system has been developed to measure visual activity to address this. The developed visual activity measurement system measures blink rate, inter-blink interval, and pupil diameter. Measured eye activity was recorded during 1 h smartphone usage of e-book reading, video watching, and social-media reels (short videos). Social media reels show increased screen variations, affecting pupil dilation and reducing blink rate due to continuous screen brightness and intensity changes. This reduction in blink rate and increase in inter-blink interval or pupil dilation could lead to visual fatigue.

## 1. Introduction

Smartphones combine computer features with basic phone functionality, a touchscreen interface, internet connectivity, and diverse applications. Smartphone usage increased by 70% during the COVID-19 pandemic due to the ease of internet access [1]. By 2023, 68.5% (5.48 billion) [2] of the world’s population owned a smartphone, and 64.6% (5.18 billion) have internet access [3]. India, the second-highest smartphone and internet user (659 million active users), experienced a significant increase in smartphone usage, with a 46.5% adoption rate by 2023 [4]. Indian smartphone users spend an average of 7 h and 18 min daily on many applications like social media, browsing, and content creation [5].

Continuous and uninterrupted smartphone use above 20 min is considered prolonged smartphone usage [6]. This prolonged smartphone usage over time can lead to physical and mental health issues, causing psychophysiological disorders [7,8,9]. Smartphone usage can also lead to digital eyestrain, with symptoms like dry eyes, blurred vision, and headaches [10,11,12,13]. Screens emitting blue light can disrupt sleep patterns, resulting in insomnia and visual fatigue [14,15,16]. Measuring fatigue induced by prolonged smartphone usage is crucial in understanding and effectively reducing fatigue. Objective measurements for visual fatigue analysis include techniques such as electrooculography (EOG) [16] and eye trackers [17]. These techniques gather data on eye movements during smartphone interactions [18,19].

The majority of the studies have examined fatigue for only 3–15 min intervals during smartphone use [7,8,9,20], which may not fully capture the extent of the fatigue caused by prolonged use, in the current smartphone era. Extended smartphone usage can lead to blue light exposure, affecting circadian rhythms, damaging the lens and cornea [21]. It can disrupt melatonin production, affect sleep, and contribute to digital eyestrain [15]. A study involving 844 adults aged 18–94 found that 60% use their smartphones before sleep [21]. The study found that prolonged smartphone usage significantly affected sleep quality and time [10]. It also affects blink rate, pupil dilation, eye movement, fixation duration, and saccade length. Eye trackers, EOG, and camera-based techniques were used to analyze eye fatigue levels. Eye trackers (Tobii, Google Glasses, and Face Lab) [11,12,22,23] use infrared technology and high-resolution cameras to measure a complete eye profile. EOG measures electrical activity in the eye muscles [13]. Both require a high investment and a large experimental setup. In addition, camera-based techniques like MATLAB v2024b and open source computer vision (OpenCV v4.10.0) use segmentation to detect facial features and the eye region [15], which requires high computational power.

The current literature has identified some research gaps: (i) Several studies have investigated the fatigue due to smartphones for short durations of 3–15 min [7,8,9,14,15,16,21], which is quite less considering the current era of smartphone utilization. (ii) Limited studies have been performed on the country with the second-highest smartphone users (India). As smartphone usage patterns [24,25] and anthropometry [26] differ across various countries, flexion angles and muscular activities vary based on their demographic data. (iii) The existing (wired/wireless) methods to measure eye parameters involve high investment, high computational power, and huge experimental setups.

With the increasing dependency on smartphones in daily life, eye strain and discomfort have become common concerns. To reduce the risks of eye fatigue, measuring the visual activity and its related parameters is essential. The study’s current objective is to develop a wireless and portable visual activity measurement setup and analyzing eye fatigue during 1 h smartphone usage. The experimental study was performed on human participants for 1 h smartphone usage for e-book reading, video watching, and social media reels.

## 2. Materials and Methods

To understand the prevalence and risks of eye fatigue due to smartphone usage, eye fatigue needs to be measured. This section outlines a detailed explanation of the developed system architecture, experimental methods, and image processing tools employed. It further elaborates the procedures followed for participant selection, the process of data collection, and the subsequent techniques used for data processing.

### 2.1. Experimental Methods and Tools

**Figure 1:**
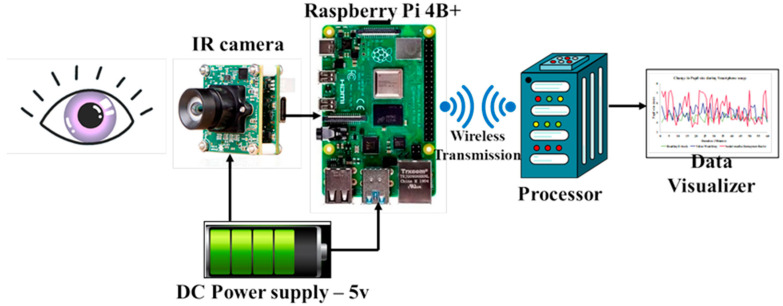
Architecture of the visual activity measurement system.

**Figure 2:**
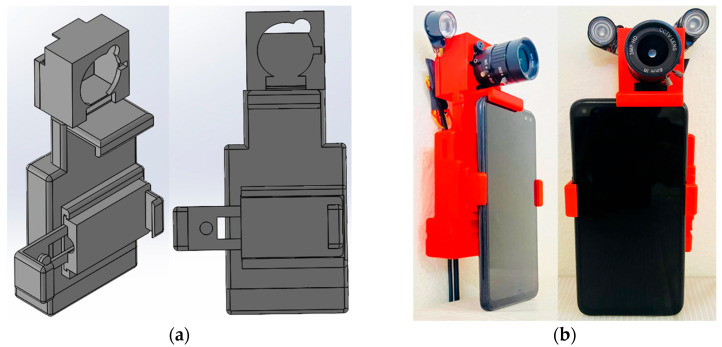
Developed camera setup and smartphone holder: (**a**) 3D design and (**b**) 3D print.

A visual activity measurement system has been developed to measure eye parameters during smartphone usage. The system includes a Raspberry Pi, an infrared (IR) camera, a power source, and a camera holder as illustrated in Figure 1 and Figure 2. The Raspberry Pi handles data collected by the camera and stores the data in an Excel spreadsheet for analysis. The IR camera captures vital eye parameters like blink rate, inter-blink interval, and pupil diameter. A portable power bank powers the transmitter part of the system.

The camera and smartphone holder assembly ensures stability and precise alignment for data capture. The system wirelessly transmits recorded data to a receiver for real-time assessment. The camera holder design is developed using SolidWorks v2024 as presented in Figure 2a, focusing on positional accuracy, ease of use, and adaptability to multiple smartphone screen sizes. The holder was fabricated using 3D printing technology as shown in Figure 2b, providing a lightweight and durable structure. The system is non-invasive, allowing participants to engage in smartphone usage naturally while capturing real-time data. The dual-purpose design of the camera holder and smartphone stand simplifies the measurement process.

### 2.2. Image Processing Techniques

**Figure 3:**
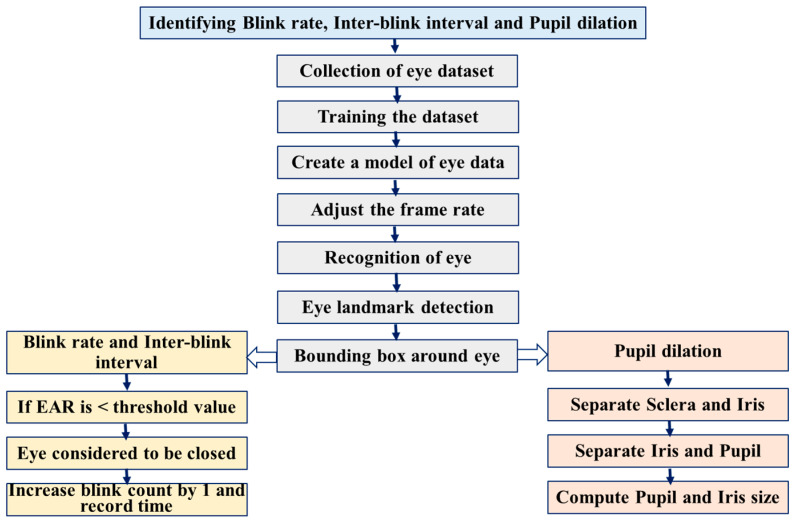
Flowchart describing the capturing of the image and its processing techniques.

**Figure 4:**
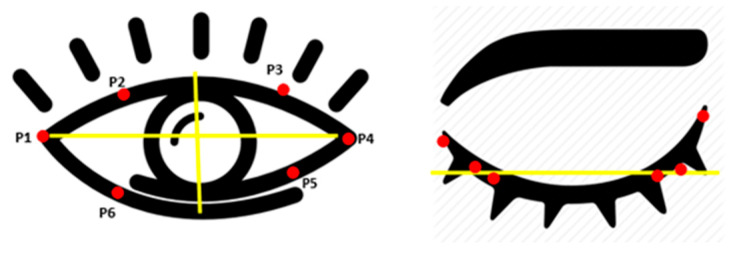
Eye aspect ratio measurement.

The developed algorithm uses facial landmark detection and the OpenCV module to accurately localize eyes and compute essential eye parameters from video streams, as shown in Figure 3. During real-time monitoring, it measures critical parameters like blink rate, inter-blink interval, and pupil dilation. The eye aspect ratio (EAR) is calculated using specific facial landmarks around the eyes. The EAR decreases when a person blinks, allowing the algorithm to detect each blink automatically. The EAR is computed based on changes in the vertical and horizontal distances between these landmarks, as shown in Equation (1) and Figure 4 [17,27]. The algorithm also monitors pupil diameter changes, providing insights into pupil dilation over time. The system is wireless, precise, and cost-effective, making it suitable for real-time applications. Its potential applications extend across human–computer interaction, healthcare, and psychology, enhancing user experience, monitoring visual fatigue, and studying cognitive responses and stress levels through eye behavior. The system offers an affordable and scalable solution for accurate, real-time eye monitoring.

(1) $$Eye\;aspect\;ratio\;(EAR)=\frac{\left|P_{2}-P_{6}\right|+\left|P_{3}-P_{5}\right|}{2\times \left|P_{1}-P_{4}\right|}$$

### 2.3. Measurement of Eye Parameters

**Figure 5:**
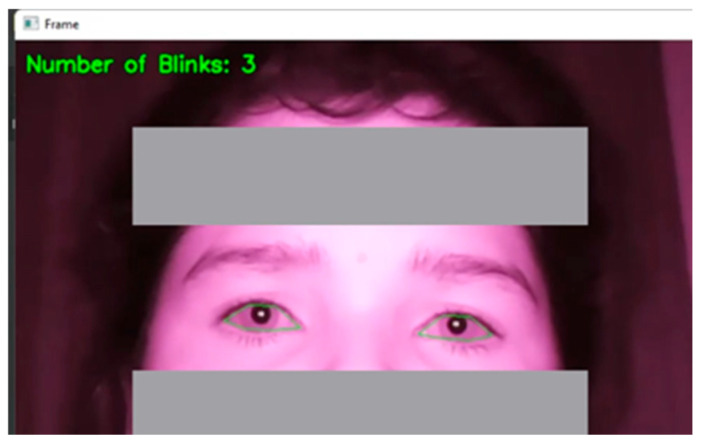
Blink rate measurement.

**Figure 6:**
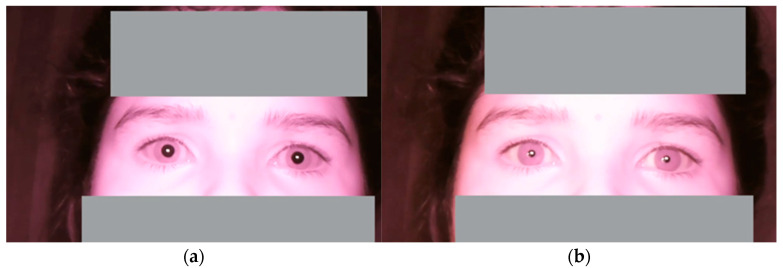
Pupil size variations: (**a**) larger and (**b**) smaller.

To measure blink rate and inter-blink interval, the system calculates the EAR. The eye is considered closed when the EAR value drops below a predefined threshold, and a blink is recorded, as presented in Figure 5. Each time the EAR reaches this threshold, the blink count is incremented by one, and the time duration between each blink is noted to calculate the inter-blink interval. Similarly, the system first separates the sclera (white part of the eye) from the iris to measure pupil dilation. Then, the system detects the iris and measures its diameter along with the pupil. The change in pupil diameter is tracked over time by comparing each frame in the video stream as presented in Figure 6a,b. This measurement provides insights into how the pupil size varies due to external factors like screen brightness or cognitive load during smartphone usage.

### 2.4. Questionnaire Assessment

Prior to the experimentation, a questionnaire was administered to gather insights into participants’ smartphone usage habits, preferred postures, the types of content they frequently accessed, and their demographic information. The questionnaire included 37 questions, and 110 participants responded. The goal was to assess smartphone usage patterns and the associated discomfort experienced during prolonged usage. The results revealed that 95% of participants used their smartphones for more than 2 h per day. The most frequently accessed content is social media (90%), texting (54%), and video watching (49%). The most commonly preferred posture during smartphone use was sitting (49%), suggesting a high tendency toward sedentary smartphone usage. Interestingly, 98% of the participants reported using their smartphones before going to sleep, a behavior that aligns with trends of excessive late-night screen time.

Regarding discomfort, 60% of participants experienced mild to severe discomfort after prolonged smartphone use, including symptoms like eyestrain, neck pain, and hand fatigue. Furthermore, 83% of the respondents indicated experiencing some form of psychophysiological disorders, such as anxiety, sleep disturbances, or mental exhaustion. To reduce discomfort, 40% of participants reported taking precautions, such as using blue light filters or enabling dark mode settings to reduce the impact of screen exposure. These findings are consistent with previous studies that highlighted the growing concern of smartphone addiction, particularly among young adults [28,29]. In this study, 30% of the participants were found to engage in excessive smartphone usage, with continuous screen time of more than 4 h spent on activities such as gaming, social media, and web browsing. This continuous smartphone use exceeding 4 h has been linked to problematic usage patterns [26,27,29]. These results underline the importance of ergonomic practices and awareness to reduce the adverse effects of prolonged smartphone use on physical and mental well-being.

### 2.5. Procedure for Selection of Participants

This study involved 30 young adults with at least one year of smartphone usage experience. The demographic data of the participants is presented in Table 1. Eye parameters such as blink rate, pupil dilation, and inter-blink interval were measured using a specialized visual activity measurement device during prolonged smartphone use. All participants provided personal information and signed an informed consent form, following the ethical guidelines. The Institutional Ethics Committee (Human Studies) of SRM Medical College Hospital and Research Centre has reviewed and approved the study design (1980/IEC/2020).

### 2.6. Experimental Procedure and Setup

The experiment analyzed the impact of different visual stimuli on eye parameters in three smartphone content categories: reading e-books, watching videos, and scrolling through social media reels. E-book reading (19.1%) required sustained focus and frequent eye movements due to black-colored text on a white background. Video watching (49.1%) [Tom and Jerry cartoon episodes] required visual and auditory stimuli, leading to changes in attention and constant brightness levels. Social media reels and short videos (90%) demanded rapid focus shifts due to the fast-changing content. This provided insights into the effects of brief visual stimuli on blink rate and pupil dilation. The continuous changes in brightness levels or content type in social media reels helped researchers understand human visual perception and behavior under different digital stimuli [17,30].

**Figure 7:**
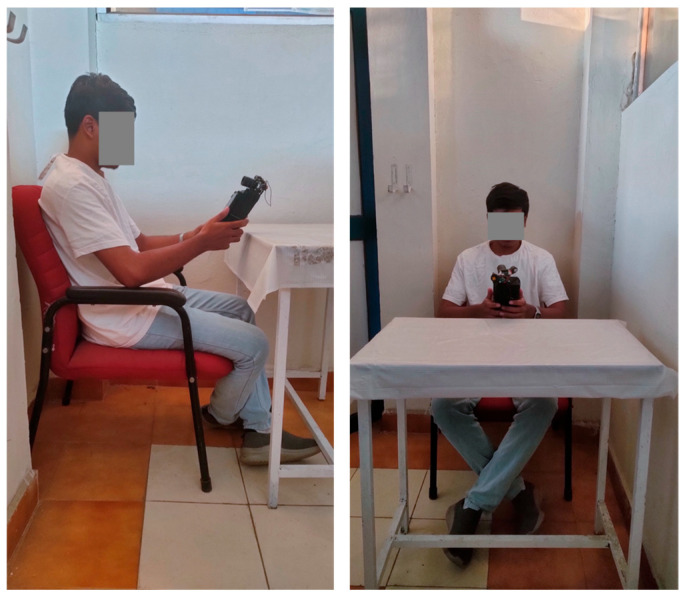
Sitting posture for the experimentation.

The study involved participants using a Real-Me 6 Pro smartphone for 60 min, with an optimal eye-screen viewing distance of 25–27 cm and 75–80 lux brightness level in the experimental room [16], as shown in Figure 7. The subjects were seated in a non-adjustable office chair (59%), with their hands resting on the armrests. The smartphone was handled in portrait mode and flight mode, with all electronics turned off to prevent interference. The study aimed to minimize external variables, allowing changes in eye parameters to be attributed solely to the content type and duration of smartphone use. This approach provided valuable data on the effects of prolonged digital screen exposure, contributing to a better understanding of visual fatigue and eye strain associated with smartphone activities. Participants were not allowed prolonged digital medium interaction before the experiment.

**Figure 8:**
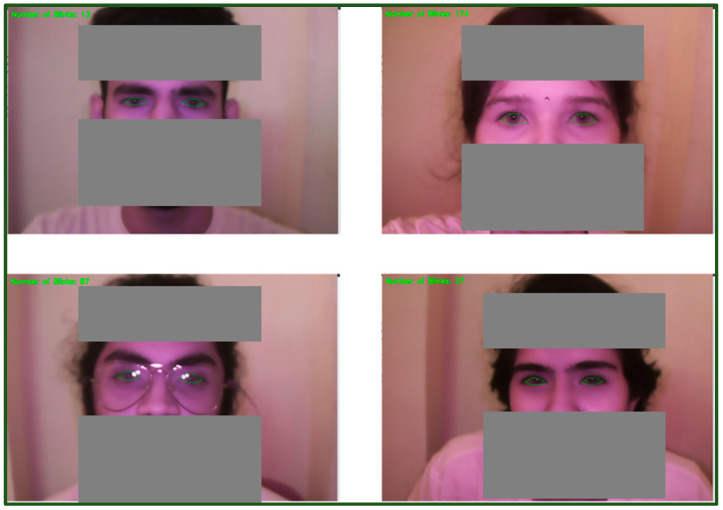
Retrieval of visual activity data (IR images).

The experiment involved participants recording their anthropometric data on day one, followed by a 60 min smartphone usage experiment (task 1) involving e-book reading, video watching, or social media reels, assigned in a random order. On the subsequent two days, participants completed additional 60 min smartphone usage experiments (task 2 and task 3), with each task involving a different content type. Figure 8 illustrates the retrieved IR images during experimentation for analyzing visual activity.

### 2.7. Analysis of Data

A statistical analysis was conducted to assess the impact of smartphone usage duration and content type on eye parameters such as blink rate, inter-blink interval, and pupil dilation. A one-way r-ANOVA [31] was used to determine significant differences in the measured eye parameters. The Shapiro–Wilk test was used to confirm normality of the variables and this ensures the appropriateness of the repeated measures analysis. Mauchly’s test of sphericity was performed. However, a significant violation of sphericity was detected, with a *p*-value of <0.05, indicating that the assumption of equal variances of the differences between conditions was not met. As a result, the null hypothesis of sphericity was rejected. To address the violation, the F-value was adjusted using the Greenhouse–Geisser correction. This adjustment provides more accurate *p*-values by accounting for the unequal variances. Additionally, a Bonferroni post-hoc analysis was performed to further assess the significant effects of the experimental factors (duration and content type) on eye parameters. All statistical computations were performed using SPSS 25.0 software for rigorous and reliable data analysis.

## 3. Results and Discussions

The participants’ average blink rate during smartphone reading, video watching and social media at different durations is presented in Table 2. Blink rates (blinks/min) varied during reading e-book (13.58 ± 2.63), video watching (12.35 ± 1.86) and social-media reels (13.33 ± 1.78) of 1 hr usage each. The average blink rate was significantly reduced (*p* < 0.05) by 61%, 54% and 59% during reading an e-book, video watching, and social media, respectively, from 0–15 min to 45–60 min in a 1 hr smartphone usage duration. This significant reduction in blink rate (*p* < 0.05) suggests that as the participants continued to use their smartphones, their blink frequency decreased, leading to potential eyestrain.

The average inter-blink interval during smartphone reading, video watching and social-media at different durations is presented in Table 3, where the inter-blink interval (sec) varied among reading e-book (3.76 ± 1.23), video watching (4.67 ± 1.61) and social-media reels (3.97 ± 2.98) of 1 hr usage each. As blink rates decreased, the inter-blink interval correspondingly increased. From the first 15 min to the last 15 min of the experiment, the inter-blink interval increased by 42% for e-book reading, 39% for video watching, and 42% for social media reels. This rise in inter-blink intervals (*p* < 0.05) is consistent with the reduction in blink frequency, indicating that participants’ eyes remained open for more extended periods as the experiment progressed.

Pupil diameter variation among the contents of e-book reading, video watching, and social media reels was 4.23 ± 1.02 mm, 4.36 ± 1.19 mm, and 5.14 ± 2.48 mm, respectively, as presented in Table 3. The average pupil diameter does not significantly change with respect to time duration. However, pupil diameter fluctuations were more noticeable during social media usage than during e-book reading and video watching. Fluctuations in pupil dilation during social media browsing stem from the dynamic reels, which have frequent changes in brightness and content. Unlike e-books and videos with steady brightness, social media’s rapid visual stimuli caused pupils to constantly adjust, increasing strain on the visual system.

**Figure 9:**
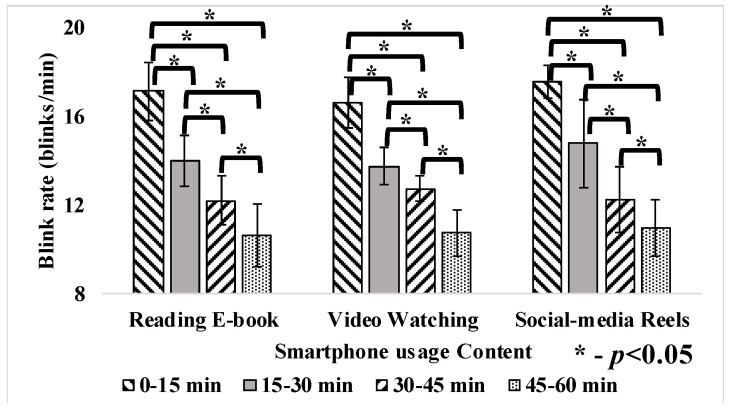
Blink rate during 1 h smartphone usage.

**Figure 10:**
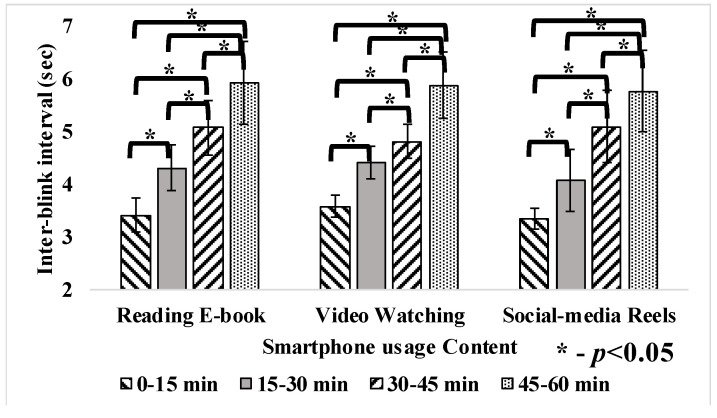
Inter-blink interval during 1 h smartphone usage.

**Figure 11:**
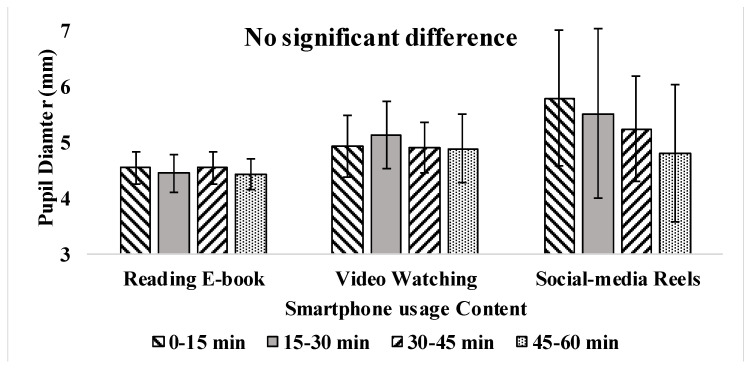
Pupil dilation during 1 h smartphone usage.

Blink rate and inter-blink interval varied significantly (*p* < 0.05) with the time duration change as presented in Figure 9, Figure 10 and Figure 11. Pupil dilation has no effect with respect to the change in time duration. However, eye parameters exhibited no significant difference with different contents as presented in Table 3. Reading e-books and watching videos did not show severe fluctuations, as the intensity and brightness of the content were almost constant. During social-media reels, variations were more in pupil dilation, as brightness and intensity of the screen changes continuously with random content. After the 40th minute, blink rate fluctuations increased with social-media reels because prolonged use leads to tired eyes, causing subjects to rub their eyes and blink hard, raising inter-blink intervals [13,16].

Previous studies align with these findings, demonstrating that prolonged smartphone usage significantly reduces the blink rate and increases the inter-blink interval. It was reported that during smartphone gaming, the blink rate reduced from 20.8 blinks to 8.9 blinks among schoolchildren [16]. Also, the inter-blink interval increased from 2.9 s to 8.7 s during smartphone usage [16]. Another study reported that the average eye blink rate varies at 18.4 blinks/min before digital medium usage. However, it reduced to 3.6 blinks/min after digital medium usage [14]. Similarly, other studies reported that the blink rate was reduced to 7 blinks/min from 22 blinks/min after office workers used any digital device during relaxation [15]. The reduction in the blink rate significantly increases the users’ inter-blink interval [27]. An increase in pupil diameter represents eye fatigue, which affects the eye’s depth of focus [15]. Studies show smartphone content affects pupil size. Demanding tasks like gaming and video watching increase diameter, while monitoring tasks like browsing and reading do not [14]. In conclusion, the reduction in blink rate to ≤8 blinks/min and increased pupil size was considered visual fatigue [14,15,32]. Eye parameters remained stable during e-book reading and video watching, likely due to the consistent screen brightness and intensity in these activities. However, social media reels led to significantly more fluctuations in pupil dilation, as the varied and fast-paced content resulted in continuous changes in brightness and contrast. These findings suggest that the dynamic nature of social media reels places greater strain on the eyes compared to more static activities like reading or watching videos, contributing to visual fatigue.

## 4. Limitations and Future Scope

Though extensive research was performed, a few limitations were encountered in this study. From pre-subjective evaluation, it was revealed that participants used their smartphones for an average of 4 h. This research examined the effects of smartphone usage for an hour. However, many past studies [7,8,9,14,15,16] reported fatigue assessment ranging between 5 and 20 min duration. The developed visual activity measurement system can only measure eye parameters (blink rate, inter-blink interval, pupil diameter) under a controlled environment. Also, the visual fatigue study is performed with only three contents of reading an e-book, video watching and social-media reels on 30 subjects for only 1 h smartphone usage duration. This sample size and duration are relatively limited compared to the widespread and extended usage of smartphones with varied content in the current era. In future studies, the fatigue induced by smartphone usage can be analyzed over longer durations to gain a more accurate understanding of the visual effects caused by different screen sizes and content types. Additionally, a broader age range and a larger sample size will be considered to improve the generalizability and robustness of the findings.

## 5. Conclusions

Prolonged use of digital devices contributes to various visual-related problems, such as eyestrain and fatigue. To reduce these issues, monitoring and measuring visual activity accurately are essential. However, existing methods for tracking eye parameters are often expensive, complex, and difficult to implement due to their bulky nature. To address these challenges, a portable and cost-effective system was developed in this study to measure key eye-related parameters, including blink rate, inter-blink interval, and pupil diameter. The study revealed that social media reels, in comparison to e-book reading and video watching, exhibit greater screen brightness fluctuations. This influences pupil dilation and reduces blink rate. The constant changes in content and brightness during social media browsing lead to more significant eye strain. Different types of smartphone content affect eye parameters, with social media reels causing more fluctuations due to varying screen brightness and intensity than e-book reading and video watching. Pupil diameter varied among the contents of e-book reading, video watching, and social media reels and exhibited values of 4.23 ± 1.02 mm, 4.36 ± 1.19 mm, and 5.14 ± 2.48 mm, respectively. Although pupil dilation did not show significant temporal changes over time, it exhibited content-related fluctuations. During social media reels, the fluctuation (standard deviation) in values from the average is nearly twice that of reading an e-book or watching videos. It causes higher variation in pupil dilation and inter-blink interval, showing that social media reels’ rapid, dynamic nature strains the eyes more than other types of content. While pupil dilation may not serve as a time-dependent marker of fatigue, it remains a relevant indicator of visual strain, as supported by previous studies. It was observed that a reduction in blink rate and an increase in the inter-blink interval or pupil dilation are indicators of visual fatigue. The results suggest that prolonged exposure to rapidly changing screen content could significantly impact visual comfort, emphasizing the need for solutions to manage screen exposure and reduce eye strain.

## Figures and Tables

**Table 1:** Subject’s demographic data (*N* = 30).

Parameters	Participants’ Data (M ± SD)
Age (years)	22.63 ± 2.83
Gender	Male = 18; Female = 12
Height (cm)	168.83 ± 6.62
Weight (kg)	71.78 ± 8.92
Eyesight (proportion)	Glasses = 68%; No Glasses = 32%
Familiarity with smartphones	>1 year
Usage of Smartphone (h/day)	5.31 ± 1.72 h

M—mean; SD—standard deviation.

**Table 2:** Statistical analysis during smartphone usage duration.

Factors	Effect–Duration	M ± SD	Effect of Duration	Effect Size	Pairwise Comparison
Blink rate (blinks/min)	0–15 min	17.33 ± 1.32	F = 49.98, *p* = 0.014	ε = 0.250; η^2^ = 0.965	Significant Difference (Steady Decrease)
	15–30 min	14.26 ± 2.01			
	30–45 min	12.35 ± 1.86			
	45–60 min	10.58 ± 1.35			
Inter-blink interval (s)	0–15 min	3.15 ± 1.62	F = 89.98, *p* = 0.031	ε = 0.457; η^2^ = 0.838	Significant Difference (Steady Increase)
	15–30 min	4.56 ± 1.23			
	30–45 min	5.94 ± 2.02			
	45–60 min	6.02 ± 1.43			
Pupil dilation (mm)	0–15 min	4.35 ± 1.62	F = 1.298, *p* = 0.219	ε = 0.923; η^2^ = 0.028	No Significant Difference
	15–30 min	4.82 ± 2.02			
	30–45 min	5.14 ± 1.93			
	45–60 min	4.92 ± 1.64			

M—mean, SD—standard deviation.

**Table 3:** Statistical analysis during smartphone usage content.

Factors	Effect– Content Type	M ± SD	Effect of Content Type	Effect Size	Pairwise Comparison
Blink rate (blinks/min)	E-book	13.58 ± 2.63	F = 1.74 *p* = 0.194	ε = 0.927; η^2^ = 0.01	No Significant Difference
	Video	12.35 ± 1.86			
	Social media	13.33 ± 1.78			
Inter-blink interval (s)	E-book	3.76 ± 1.23	F = 1.62 *p* = 0.185	ε = 0.894; η^2^ = 0.03	No Significant Difference
	Video	4.67 ± 1.61			
	Social media	3.97 ± 2.98			
Pupil dilation (min)	E-book	4.23 ± 1.02	F = 1.26 *p* = 0.179	ε = 0.963; η^2^ = 0.03	No Significant Difference
	Video	4.36 ± 1.19			
	Social media	5.14 ± 2.48			

M—mean, SD—standard deviation.

## Data Availability

The data presented in this study are available from the corresponding author upon request. The data are not publicly available due to privacy concerns, proprietary or confidential information, and intellectual property belonging to the organization that restricts its public dissemination.

## Abbreviations

The following abbreviations are used in this manuscript:

EAR	Eye aspect ratio
EOG	Electrooculogram
IR	Infrared
OpenCV	Open source computer vision
